# MANAGEMENT AND PREVENTION OF SUICIDE RISK IN PRISON: RESULTS OF A PRELIMINARY STUDY ON ITALIAN PRISONERS

**DOI:** 10.1192/j.eurpsy.2023.2351

**Published:** 2023-07-19

**Authors:** F. Cordasco, M. A. Sacco, C. Scalise, A. P. Tarallo, S. Gualtieri, P. Ricci, I. Aquila

**Affiliations:** 1 Institute of Legal Medicine University Magna Graecia of Catanzaro, Catanzaro, Italy; 2 Institute of Legal Medicine University Magna Graecia of Catanzaro, Catanzaro, Italy

## Abstract

**Introduction:**

There are about 10 million inmates in the world, of which 6 million are held in American prisons. While in Europe there was a 6.6% reduction in the detention rate, in Italy the number of prisoners is constantly growing. Due to the worldwide importance of the phenomenon in the field of public health, it is necessary to analyze the relationship between detention and prisoner health, as well as the change in the psychophysical state of a subject after a period of incarceration.

**Objectives:**

The analysis of the results deriving from the completion of forensic examinations was carried with the aim of assessing the compatibility of the health conditions of prisoners with imprisonment.

**Methods:**

We report the results of a preliminary study on a sample of fifty prisoners held in Southern and Central Italy prisons.

**Results:**

The average age of the prisoners was 41. The prevalence of psychiatric disorders in the sample examined was about 39 %. 45 % of subjects with mental disorders made one suicide attempt in prison at least. We emphasize the pathogenic role of prison in the development or aggravation of psychiatric disorders. This happens particularly in subjects coming from degraded socio-family backgrounds.

**Conclusions:**

In order to reduce the prevalence of psychiatric disorders and the risk of suicide, it is necessary to carry out careful medical and psychological evaluation at each new entry, so as to be able to frame the inmate’s state of health and plan periodic monitoring of diagnosed diseases. This evaluation should include a psychiatrist, so as to be able to set up an adequate drug treatment when it’s necessary. The correct management of psychiatric disorders is essential in order to improve the inmate’s mental health and prevent medico-legal consequences for health workers.

**Disclosure of Interest:**

None Declared

